# Role of Hypoxia-Mediated Autophagy in Tumor Cell Death and Survival

**DOI:** 10.3390/cancers13030533

**Published:** 2021-01-30

**Authors:** Rania F. Zaarour, Bilal Azakir, Edries Y. Hajam, Husam Nawafleh, Nagwa A. Zeinelabdin, Agnete S.T. Engelsen, Jérome Thiery, Colin Jamora, Salem Chouaib

**Affiliations:** 1 Thumbay Research Institute for Precision Medicine, Gulf Medical University, Ajman 4184, UAE; dr.rania@gmu.ac.ae (R.F.Z.); husam@gmu.ac.ae (H.N.); nagwa@gmu.ac.ae (N.A.Z.); 2 Faculty of Medicine, Beirut Arab University, Beirut, Lebanon; b.azakir@bau.edu.lb; 3 IFOM-inStem Joint Research Laboratory, Institute for Stem Cell Science and Regenerative Medicine (inStem), Bangalore, Karnataka 560065, India; yousafh@instem.res.in (E.Y.H.); colinj@instem.res.in (C.J.); 4 School of Chemical and Biotechnology (SCBT), Shanmugha Arts, Science, Technology and Research Academy (SASTRA), deemed to be University, Thanjavur 613401, Tamil Nadu, India; 5 Department of Biomedicine, Centre for Cancer Biomarkers CCBIO, University of Bergen, 5007 Bergen, Norway; Agnete.Engelsen@uib.no; 6 INSERM UMR 1186, Integrative Tumor Immunology Immunotherapy, Gustave Roussy, Faculty of Medicine University of Paris-Sud, University of Paris-Saclay, F-94805 Villejuif, France; jerome.thiery@gustaveroussy.fr

**Keywords:** hypoxia, apoptosis, inflammation, cell survival, stemness, tumor resistance

## Abstract

**Simple Summary:**

Autophagy is a self-eating mechanism that is involved in the degradation of organelles and cellular materials. It is initiated by intracellular and extracellular stress stimuli. In the context of tumor development, microenvironmental hypoxic stress regulates autophagy that, in turn, promotes cancer-cell death or cancer-cell survival. Autophagy functions and shares molecular players with other cell-death promoting pathways such as apoptosis. Here, we discuss the spatial and temporal control of autophagy that could result in opposing cellular outcomes. We also address the role of immune cells polarization in this context. This knowledge is essential for efficiently targeting autophagy in conjunction with immunotherapy for improved cancer treatment.

**Abstract:**

Programmed cell death or type I apoptosis has been extensively studied and its contribution to the pathogenesis of disease is well established. However, autophagy functions together with apoptosis to determine the overall fate of the cell. The cross talk between this active self-destruction process and apoptosis is quite complex and contradictory as well, but it is unquestionably decisive for cell survival or cell death. Autophagy can promote tumor suppression but also tumor growth by inducing cancer-cell development and proliferation. In this review, we will discuss how autophagy reprograms tumor cells in the context of tumor hypoxic stress. We will illustrate how autophagy acts as both a suppressor and a driver of tumorigenesis through tuning survival in a context dependent manner. We also shed light on the relationship between autophagy and immune response in this complex regulation. A better understanding of the autophagy mechanisms and pathways will undoubtedly ameliorate the design of therapeutics aimed at targeting autophagy for future cancer immunotherapies.

## 1. Introduction

Cell fate decisions of whether to live or to die are tightly regulated by a complex system of balanced signaling pathways and these decisions correlate directly with health and disease. Cells need to cope with a multitude of variable intracellular and environmental stress stimuli, responses to which, are linked to cytoprotection or cytotoxicity. In this regard, the tumor microenvironment (TME), including deprivation of adequate oxygen supply, namely hypoxia, plays an important role [1,2]. Compared to normal cells, cancer cells may adapt faster to microenvironmental modifications, i.e., by activating multiple stress response pathways, and circumventing anti-proliferative signals and cell death inducing signals. In response to hypoxia, cells activate the transcription factor hypoxia-inducible factor-1α (HIF-1α) that, in turn, activates numerous hypoxia inducible genes and thus promotes angiogenesis to boost proliferation of tumor vasculature.

Hypoxia also activates additional pathways including autophagy, which is a regulated program for the degradation and recycling of cellular components that has been shown to play a crucial role in the hypoxia-induced tumor response. Autophagy was initially identified as a multistep catabolic process that promotes lysosome-mediated degradation of nonessential or damaged cellular constituents [3]. Briefly, the process of autophagy is initiated by the formation of phagophores, followed by the accumulation of autophagosomes and the degradation of cargo by acid hydrolases in autolysosomes, which is the organelle formed by the fusion of autophagosomes and lysosomes. Autophagy involves five key regulatory steps, namely initiation, nucleation, elongation, lysosomal fusion and degradation, and each step is regulated by a series of protein complexes that modulate the autophagic activity [4,5,6].

Oxygen deprivation is considered the most established stimulus for the induction of autophagy [7]. The net outcome of hypoxia-induced autophagy in the TME is complex and controversial. Recent evidence suggests that autophagy is a double-edged sword that may induce either cell death or cell protection. By clearing damaged organelles and molecules, autophagy is involved in maintaining healthy cell function and protects the organism against tumor development [8,9]. However, once the malignancy has developed, autophagy may promote cancer cell survival and growth. Furthermore, autophagy allows reprogramming of the tumor microenvironment and confers to the tumor the ability to become resistant or sensitive to chemotherapy-induced toxicities [10]. As such, inhibition of autophagy has recently evolved as a strategy to enhance the efficacy of chemotherapy [11]. Indeed, autophagy can have both pro and anti-tumor activities according to the cancer stage, time, and extent of the ischemia [12].

Recent evidence has shed light on the processes involved in the regulatory role of autophagy during hypoxia. Autophagy promotes cancer cell survival by producing sufficient ATP via the recycling of free amino acid and free fatty acids, and thus helps tumor cells overcome necrosis and apoptosis. Autophagy is also intimately associated with cell death. Indeed, autophagic cell death has been characterized as type II of the three identified forms of programmed cell death, apoptosis and necrosis being type I and type III, respectively [13].

A better understanding of the molecular mechanisms underlying the complex dual role of autophagy as a determining factor in the field of cancer biology, cancer immunology and immunotherapy is needed. Thus, it is crucial to elucidate the functional consequences of autophagy in shaping the stroma reactivity, reprogramming the tumor microenvironment, and modulating tumor heterogeneity, all in the context of the enormous cellular plasticity in cancer. This will be of major importance in order to integrate autophagy induction or targeting in future cancer therapy approaches.

## 2. Microenvironmental Hypoxic Stress Induced Autophagy

### 2.1. As a Survival Mechanism for Hypoxic Cells

The most common stimuli that induce autophagy are oxygen deprivation and nutrient scarcity. Under hypoxic stress, the transcription factor HIF-1α is stabilized and thus activates the expression of multiple genes involved in signaling pathways that maintain oxygen and energy homeostasis [14]. Three isoforms have been identified for the HIF-α family, namely HIF-1α and HIF-2α, that are responsible for the hypoxia mediated cell responses, and HIF-3α, of which less is known [15].

**Figure 1:**
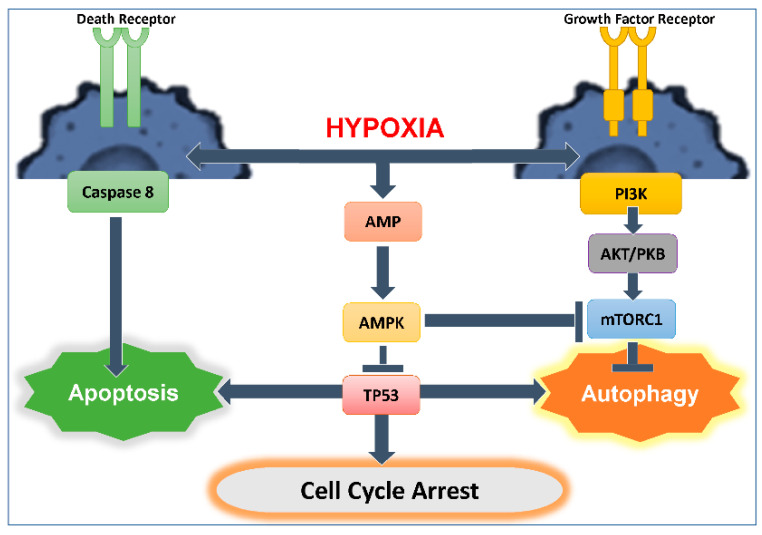
A model of hypoxia-induced autophagy and cell death. Inactivation of mTOR during hypoxia leads to the initiation of autophagy. Hypoxia inactivates mTOR downstream of PI3K and AMP pathways. On the other hand, hypoxia causes Caspase 8 activation and subsequent apoptosis. TP53 plays a key role at the center of these pathways where it can cause cell cycle arrest, and promote autophagy or apoptosis.

Hypoxia-mediated autophagy has been extensively studied; however, the exact signaling pathways underlying the role of HIF-1α remain elusive [15,16]. HIF-1α is implicated in regulating the expression of key genes involved in the initiation and progression of autophagosomes formation, including Bcl-2, adenovirus E1B 19 kDa-interacting protein 3 (BNIP3), Beclin 1, BNIP3-like (BNIP3L)/NIX, Phosphatidylinositol 3 kinase catalytic subunit type 3 (PI3KC3), ATG7, ATG5, and ATG9A [17,18,19,20,21,22]. Mammalian target of rapamycin complex 1 (mTOR C1) is a Ser/Thr kinase that controls cell growth [23]. mTOR is the major downstream effector of the phosphoinositide 3-kinase (PI3K)/Akt signaling pathway [24]. The activity of mTOR is inhibited under nutrient starvation or oxygen deprivation [25,26], an essential step for autophagy induction (Figure 1). Moreover, autophagy can be promoted through ULK1 phosphorylation during energy or nutrient loss by activated AMP-activated protein kinase (AMPK). However, autophagy may itself also regulate HIF-1α stability, and this might partially explain the opposing roles of autophagy in malignant tumors [27].

**Figure 2:**
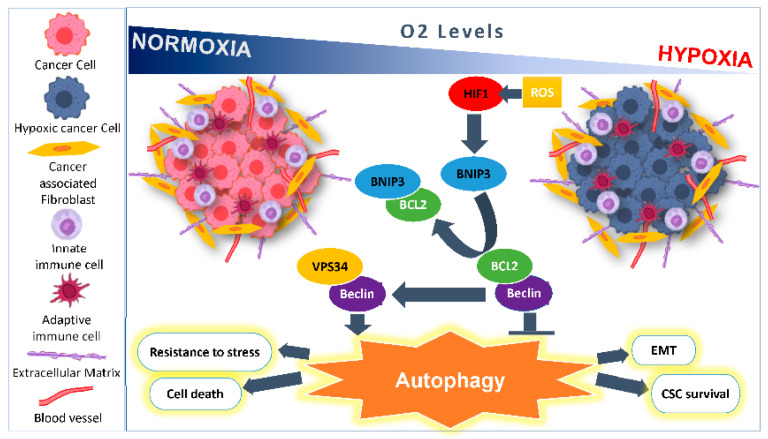
Autophagy induced by hypoxia promotes cancer cell survival. Hypoxia-inducible factor HIF-1 promotes transcription of BNIP3, thereby activating Beclin-1 by disrupting the Bcl-2-Beclin-1 complex. Free Beclin-1 associates with VPS34 and results in the activation of autophagy. Autophagy functions in both helping cells resist stress, by promoting EMT and CSC survival, and induction of cell death. The cytotoxic effects of the immune cells that infiltrate the tumor microenvironment are attenuated by autophagy.

The impact of HIF-1α on autophagy occurs through BNIP3 and Bcl-2 regulation. HIF-1α has been shown to induce autophagy by increasing the expression of both BNIP3 and Beclin proteins in lung cancer cells, leading to cisplatin resistance [28]. The interaction between Beclin-1 and Bcl-2 is regulated by BNIP3 and BNIP3L/NIX, both transcriptionally induced under hypoxia, and can disrupt the inhibitory interaction between Bcl-2 and Beclin-1, due to the higher affinity of Bcl-2 to BNIP3 [29]. Therefore, BNIP3 renders Beclin-1 free to form a complex with VPS34 that is otherwise inactivated in the presence of Bcl-2, and subsequently promotes the nucleation of isolated membranes [29] (Figure 2). Hypoxia can further enhance the expression of BNIP3 by Ras and E2 transcription factor (E2F) which, in turn, can be reduced by the activity of both retinoblastoma protein (Rb) and NF-kB [30,31,32,33]. Studies showed that silencing the expression of BNIP3 and BNIP3L/NIX completely inhibits autophagy induction by hypoxia in CCL39 cells and renders them pro-apoptotic proteins [34,35]. In addition, these cells become sensitive to etoposide-induced apoptosis under hypoxic condition [36]. Similarly, BNIP3 induces mitochondrial dysfunction and promotes autophagy and apoptosis in neonatal cardiac myocytes under hypoxia [37]. Thus, BNIP3 and BNIP3L/NIX proteins are essential inducers of autophagy in response to hypoxia. Both BNIP3 and BNIP3L/NIX are found to be overexpressed in carcinoma cells of various origins including breast cancer under hypoxic induction [15], and high expression of BNIP3L correlates with shorter disease-free survival time [38]. In contrast, BNIP3 was not found to be expressed in other cancer cell types including pancreatic cancer, gastric cancer, multiple myelomas, and primary colorectal cancers (CRCs), rendering these cells more prone to resist apoptosis [39,40], and a decrease in BNIP3 expression has been shown to lead to poorer survival and cell proliferation in renal cell carcinoma (RCC), as well as pancreatic and CRCs [41,42,43].

Together, these results point to an inconsistent correlation between BNIP3 and HIF1 expression in several cancers [44]. This is further demonstrated in RCC tumor tissue where BNIP3 and VHL expression levels are lower compared to adjacent non-tumor tissues, whereas the expression of HIF-1α was higher in the same tumor tissues. Epigenetic regulation of BNIP3 further adds to the complex regulation of this gene. In RCC, acetylation of the BNIP3 gene results in an increase in its expression as well as inhibiting cell proliferation [42]. This is in contrast to results in hepatocarcinoma where demethylation and not acetylation of BNIP3 promoter restores its expression [45]. Even though the role of BNIP3 in regulating hypoxia inducing autophagy is established, hypoxia inducing BNIP3 expression may not be involved in the induction of autophagy [46] and autophagy may also be induced independently of HIF-1α expression [47,48]. These results indicate that there is delicate balance contributing to cell survival and cell death, making it exceedingly difficult to determine the stage at which targeting BNIP3 may be of benefit for tumor death and patient survival.

During hypoxic conditions, cells must adapt to the consequences of reduced oxygen availability, which has a direct negative impact on the cell’s capacity to produce ATP and maintain energy homeostasis. Another important role of HIF-1α and autophagy in this context is to increase the ability of the hypoxic cancer cells to activate signaling pathways that promote ATP production independently of mitochondrial oxidative phosphorylation. HIF-1α induces increased glucose uptake, lactate production and reduces oxygen consumption and production of reactive oxygen species (ROS) [49]. This synergetic activity enhances the production of ATP and pyruvate and induces anaerobic glycolysis. HIF-1α induces the expression of glucose transporters (GLUTs), and autophagy regulates the uptake of glucose by increasing the plasma membrane translocation and activity of GLUT1 [50]. However, during hypoxia, cells cannot maintain adequate antioxidant capacity, resulting in increased ROS levels. ROS subsequently promotes mitochondrial outer membrane permeabilization (MOMP) process and increases the expression of the pro-apoptotic proteins Bax/Bcl-2, Bax/Bcl-xL ratio, poly-(ADP-ribose)-polymerase (PARP) fragments and caspases, leading to the eventual activation of apoptosis [51]. Moreover, high levels of ROS may induce autophagy mediated cell protection. In fact, autophagy reduces ROS levels through eliminating damaged mitochondria, through a process called mitophagy that maintains a healthy and functional mitochondrial pool. Thus, autophagy eliminates ROS and ROS damaged proteins, reduces cell damage and inhibits cell death, and enhances cell survival mechanisms, as it was shown in myocytes and kidneys subject to ischemia/reperfusion (IR) [52,53]. Moreover, cells treated with ROS scavenger (NAC) reduce both ROS levels and autophagic flux [54]. Importantly, on the one hand, this negative feedback between autophagy and ROS protects cells from oxidative damage and ultimately from death [55]. On the other hand, however, HIF-1 activation is regulated by ROS, which mediates HIF-1 nuclear translocation and stabilization and, in turn, triggers the expression of HIF dependent genes, including BNIP3/NIX [56].

### 2.2. Autophagy Promoting Immunosuppression

There is a plethora of evidence available on the role of autophagy in tumor evasion from immune surveillance [57]. For example, M2 Macrophages cause immune suppression and thus help the tumor escape from immune surveillance. Upon inhibition of autophagy in tumor cells, the release of TRAPs (tumor cell-released autophagosomes) is prevented. TRAPs can cause the polarization of macrophages towards M2 like phenotype, thus promoting immunosuppression [58]. In the mouse model of breast cancer, inhibition of autophagy by knockout of the focal adhesion kinase family interacting protein of 200 kDa (FIP200) decreases tumor progression and increases the infiltration of anti-tumor CD8 + T cells [59]. Similar results of autophagy inhibiting CD8 + T recruitment were observed in lung cancer models [60]. Further evidence comes from human melanoma cells where autophagy inhibition led to a decrease in tumor growth and an increase in accumulation of anti-tumor T cells [61] and Natural killer cells [62]. Regulatory T (T regs) cells exert their immune suppressive functions on other immune cells and hence maintain tolerance for tumors; upon deletion of ATG7 from T regs, the suppressor function was compromised and anti-tumor CD8 + T cells infiltrated, which resulted in the inhibition of tumor growth [63]. Lastly, autophagy inhibition in Myeloid-derived suppressor cells (MDSC) enhances MHC II expression and T cell activation, and, at the same time, reduces tumor growth [64]. These results indicate that autophagy plays a significant role in helping the tumor cells to evade immune surveillance.

### 2.3. Autophagy Promoting Tumor Resistance to Immune Cell-Mediated Cytotoxicity

During the anti-tumor response, malignant cells are eradicated by the cytotoxic machinery of the immune system including natural killer (NK) cells and cytotoxic T cells (CTLs). Accumulating evidence indicates that the hypoxic microenvironment contributes to cancer cell resistance to immune cell mediated killing, which can be detrimental to anti-tumor effector cell functions. In previous studies, we provided evidence indicating that the quality of the adaptive and innate immune or NK cell cytotoxic responses and survival pathways are shaped by autophagy; subsequently, this may impact the clinical benefit of immune cell-based therapies. In this regard, our studies first established a functional link between the regulation of antigen-specific T-cell lysis and autophagy, and this points to a major role for autophagy in the promotion of tumor growth in vivo [60]. We have demonstrated that blocking hypoxia-induced autophagy in tumors restores cytotoxic T-cell activity and promotes regression. Very recently, Lawson et al., identified autophagy as a conserved mediator in the CTLs-evasion by cancer cells; they also showed that autophagy is required to resist IFNγ and TNF-induced cytotoxicity [65]. Nevertheless, Okamura et al., have shown that autophagy is effective in creating CTL epitopes that mimic tumor-associated antigens, suggesting that the processing of ubiquitously expressed proteins by autophagy mechanisms could contribute to the generation of specific tumor-associated antigens [66]. Akalay et al., reported that in addition to the well-characterized role of epithelial to mesenchymal transition (EMT) in cancer cell phenotypic changes that include a tumor-initiating cell phenotype, EMT-induced cancer cell resistance to CTLs mediated cell lysis, which correlates with the induction of autophagy [67].

**Figure 3:**
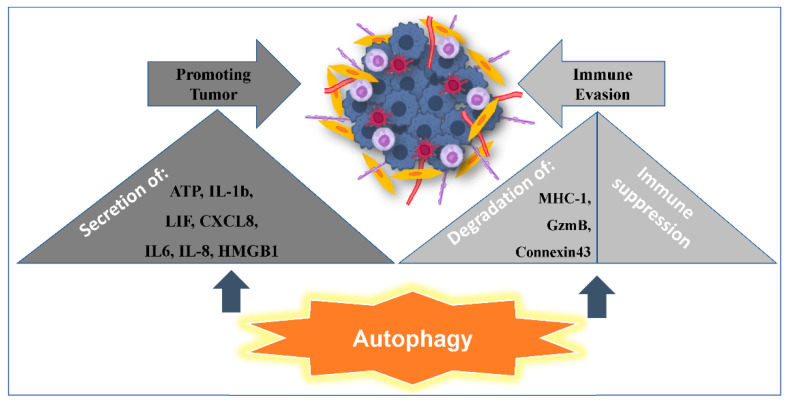
Autophagy as a double-edged sword: Autophagy induces the secretion of tumor promoting proinflammatory factors (ATP, IL-1b, LIF, CXCL8, IL6, IL-8, HMGB1) and helps in immune evasion, either by degradation of anti-tumor factors (MHC-1, Granzyme B and Connexin 43) or by promoting immune suppression.

Several studies demonstrated that autophagy is a key regulator of the innate immune response. Specifically, Janji et al., have elegantly demonstrated that the susceptibility of breast cancer cells to NK-mediated lysis is impaired by hypoxia-induced autophagy, and that this is reversed by targeting autophagy [68]. We provide evidence that the activation of autophagy in hypoxic cells blocks the NK-mediated target cell apoptosis; this is due to autophagy being involved in the selective degradation of GZMB/granzyme B, a pro-apoptotic NK-derived serine protease [68]. Similarly, it has been reported that tumor cells utilize autophagy to evade immune attack by degradation of MHC-1 [69] and Connexin 43 [70] (Figure 3). Degradation of these surface molecules prevents the formation of the immunological synapse. In a recent study, it has been demonstrated that targeting Beclin1 inhibited tumor growth; in addition, for NK-cells to infiltrate the tumor bed, they relied on CCL5 overexpression by the autophagy-defective tumors [62]. More importantly, Messai et al., demonstrated that HIF-2α led to overexpression of ITPR1, which subsequently regulated the NK-mediated killing by activating autophagy in target cells via NK-derived signal. Interestingly, NK-induced autophagy was inhibited by silencing both ITPR1 and Beclin-1, and this subsequently increased granzyme B activity in target cells [71]. Finally, we have shown a correlation between melanoma patients failing to respond to anti-PD1 (pembrolizumab) immunotherapy and increased levels of glycogen branching enzyme1 (BNIP3/GBE1) [38]. This result thus indicates that hypoxia, which elevates glycogenic flux and autophagy, is a critical molecular program that could be considered as a prognostic factor for melanoma.

### 2.4. Microenvironmental Hypoxic Stress Induced Autophagy as a Modulator of Tumor Immunogenicity and Adjuvanticity

Hypoxia may play an important role as a driver of intratumor genetic and non-genetic heterogeneity, and greatly affect the composition of the tumor immune microenvironment [72]. Microenvironmental hypoxic stress induced autophagy may benefit cancer cells through cytoprotective cancer cell intrinsic mechanisms and through increased resistance to cell-mediated cytotoxicity, as discussed above. Recent accumulating evidence also demonstrates that autophagy is directly involved in the processing and presentation of cancer cell endogenous antigen by MHC-I, and also by exogenous antigen presentation by MHC-II, on professional antigen presenting cells (Chapter 10 from Reference [73]). Thus, autophagy has been established as an important player in the dynamic process of “immunoediting” where cancers evolve to avoid immune recognition and killing [74], and autophagy is also being explored as a target experimentally in order to shift the balance from cancer cytoprotection to “immunosurveillance”. Although much is yet to be learned about the role of autophagy in the context of cancer therapy (Chapter 8. From Reference [73]), the high-pre mortem autophagy of tumors, in particular, may eventually be exploited to aid in the process of immune cell infiltration through increased release of immune stimulatory signals.

In order to initiate the immune response to effectively eradicate cancer cells, multiple sequential steps described by Chen and Mellman as the “cancer immunity cycle” must be fulfilled [75]. Recently, the role of autophagy in the initial step of the cancer immunity cycle has received a lot of attention in the cancer research community. In this step, professional antigen-presenting cells (APCs) capture and release cancer neo-antigens. In brief, upon encountering cancer neo-antigens, the professional APCs exit the tumor microenvironment and travel to the lymph node where they present the cancer antigens to the T-cells via their MHC molecules. This process educates T-cells to recognize and act upon the presented epitope of the cancer neo-antigen and activates the T-cells. In the subsequent step, the activated T-cells enter the vasculature and home to the tumor site where they infiltrate the tumor microenvironment and may finally recognize and eliminate the neoantigen expressing cancer cells [75]. In order to initiate the cancer immunity cycle successfully, the expression of cancer-associated antigens harboring the potential to be recognized by APCs (i.e., the immunogenicity) alone is not sufficient. Additional signals specifically potentiating a robust immune response (i.e., adjuvanticity) are also required for robust initiation of the “cancer immunity cycle” [76,77]. Thus, the ultimate goal of cancer immunotherapy is to enable re-activation or de-repression of a halted cancer immunity cycle through targeting and increasing the efficiency of each step of the “cancer immunity cycle” [78]. Increased release of danger/damage-associated molecular patterns (DAMPs), which act as immunoadjuvant signals in the tumor immune microenvironment and exert their effect by bridging the innate and adaptive arms of the immune system, has been linked to high pre-mortem autophagy. In brief, DAMPs include “find me” signals, such as the release of chemokines or ATP into the extracellular space, and the exposure of “eat-me” signals, including relocation of phosphatidylserine to the outer leaflet of the cell membrane as well as cell surface externalization of calreticulin (CALR).

Upon extracellular exposure in the tumor immune microenvironment, DAMPs potently activate the immune system through the binding of pattern recognition receptors (PRRs) [79]. DAMPs are released by the cells as endogenous danger signals in response to cell death or various modes of excessive cellular stress, including high pre-mortem autophagy. Upon the subsequent activation of the DAMP receptors (including toll-like receptors (TLRs) and the receptor for advanced glycation and products (RAGE)), multiple intracellular signaling pathways are activated, including mitogen-activated protein kinase (MAPK), NF-κB, and phosphatidylinositol 3-kinase (PI3K)/AKT signaling pathways [79]. Of note, although the relationship between the released DAMPs and autophagy in response to cellular stress or injury is complex and seems to be highly context-dependent, it is considered likely to be important in regulating cancer progression and also modulating the potency of anti-tumor treatments, and thus this issue deserves further attention to clarify when and how autophagy could be targeted to increase adjuvanticity in the context of improving cancer immunotherapy [80]. While still largely unexplored, the interdependent regulation seems to be based on a finetuned molecular crosstalk where autophagy, on the one hand, regulates the release as well as the degradation of DAMPs, and on the other hand, autophagy itself may be triggered by the release of DAMPs [81]. While increasing evidence reveals that pre-mortem autophagy is important for the release of DAMPs, as discussed in detail in our recent review (Chapter 8 from Reference [73]), autophagy itself may also represent an attractive target in other clinical settings, for example, in oncogene-addicted cancers and in less immunogenic cancers where the autophagy mediated adjuvanticity is not expected to add substantially to the therapeutic benefit.

## 3. Autophagy and Apoptosis Relationship

### 3.1. Interplay between Autophagy and Apoptosis

Autophagy is generally considered a cell survival mechanism; it is a cell’s attempt to cope with stress. Autophagy and apoptosis often take place in a sequence where autophagy precedes apoptosis. Indeed, numerous reports have implicated autophagy in the regulation or induction of cell death [82]. In certain situations, autophagy can help to both induce apoptosis and process crosstalk through interconnecting signaling pathways [83,84]. Nevertheless, the outcome of this regulation is still controversial and the discrepancy in the outcomes can be correlated with a significantly different response observed between normal and cancer cells.

As such, Beclin-1, an activator of cellular autophagy, is at the crossroad between autophagy and apoptosis through direct interaction with anti-apoptosis family members, including Bcl-2 and Bcl-X_L_ [85]. Consequently, several BH3 (Bcl-2 Homology 3) only proteins, including Bad, Bid, BNIP3, Noxa or Puma, well known to promote apoptosis, can also promote autophagy by disrupting the inhibitory interaction between Beclin-1 and Bcl-2 or Bcl-X_L_. Another example highlighting the crosstalk between autophagy and apoptosis is the capacity of Ser/Thr kinases (including Akt and JNK) to regulate both phenomena. In particular, JNK can trigger both apoptosis and autophagy by phosphorylating Bcl-2 in its flexible loop between BH4 and BH3 domains, which decrease its inhibitory activity on Beclin-1 and on pro-apoptotic members of the Bcl-2 family [82]. Although BH3-only proteins stimulate apoptosis and autophagy, the exact sequential mechanism depends on the intensity and duration of the stimulus. At low stress level, BH3-only proteins promote Beclin-1 interaction with VPS34 to initiate autophagy. On the contrary, at high stress level, BH3-only proteins have been shown to shift the cell response toward apoptosis by activating the caspase cascades [86].

Autophagy can inhibit apoptosis by multiple mechanisms. The first mechanism is by upregulating mitophagy. Mitophagy is induced by the loss of mitochondrial membrane potential (ΔΨm), leading to increased inner membrane permeability and the ubiquitylation of multiple outer membrane proteins such as voltage-dependent anion-selective channel 1 (VDAC1), mitofusin 1 (MFN1) and MFN2 promoting mitophagy [87,88,89]. Mitophagy induction has been observed by an increasing accumulation of P62 in the pre-ischemic cortex of a mouse model of transient middle cerebral artery occlusion (MCAO) treated with rapamycin [90]. Thus, rapamycin could attenuate ischemic brain injury via the induction of mitophagy. Moreover, ATG7 knock down or treatment with 3-methyladenine (3-MA) inhibits autophagy and leads to the accumulation of damaged mitochondria in the cortex of MCAO mice, the release of Cytochrome C and the activation of apoptosis [91]. These results demonstrate that hypoxia-induced autophagy can prevent apoptosis by degrading the mitochondria, thus inhibiting Cytochrome C release and subsequent apoptotic cascade activation.

Autophagy can also inhibit apoptosis independently of mitochondria. In response to hypoxia, externalized phosphatidylserine, as detected by Annexin V-FITC, increased in EAhy926 cells treated with 3-MA accompanied with an increase in caspase 3 activity [92]. Another mechanism is by inducing the degradation of apoptotic proteins including the active caspase 8 [93] or inhibitors of apoptosis (IAPs) [94]. For example, BRUCE, a member of the IAP family, has been shown to accumulate in autophagy germline mutant egg, indicating that autophagy eliminates BRUCE and reduces apoptosis [94]. In summary, autophagy activation can promote cytoprotection by inhibiting apoptotic pathway intermediates, such as by eliminating damaged mitochondria, reducing ROS levels, and blocking caspase 3 activation.

However, apoptosis can also inhibit autophagy, mainly by caspase-mediated cleavage of essential autophagy components [95]. The activation of caspases 3 and 8 causes ATG3, ATG4D and Beclin-1 cleavage and suppression of autophagy [96,97].

In contrast to the previously mentioned effect, when the cellular capacity to cope with stress is overwhelmed due to the intensity or the duration of the stimulus, autophagy can activate apoptosis. Autophagy mediates apoptosis activation by the action of caspases. Fragmented Beclin-1 can translocate to the mitochondria, causing mitochondria permeabilization and the release of Cytochrome C [98], fragmented ATG4 may require a BH3 like domain and induce apoptosis [96], and fragmented ATG5 translocates to the mitochondria and activates apoptotic signals. Moreover, inhibiting autophagy by 3-MA was shown to cause a reduction in the caspases’ activity post-ischemia, which prevents apoptosis [99]. Interestingly, inhibition of autophagy response could be related to the extent to which autophagy is blocked. Knocking out ATG3 or ATG5 blocks the early steps of autophagy and inhibits the subsequent activation of the caspases’ cascades; however, Bafilomycin A, an inhibitor of late autophagy, increases caspases’ activity and eventually apoptosis [100]. Of note, autophagy can induce cell death; however, this occurs independently of the effects of the autophagic flux, by a process known as autophagic cell death. Direct inhibition of autophagy using pharmacological compounds or by reducing or depleting one of its components prevents cell death in certain cancer cells [101,102,103].

### 3.2. Role of TP53 in Autophagy Induction and Regulation: Influence on Anti-Tumor Cytotoxic Immune Response

Hypoxic stress, among other types of stressors, including oxidative stress and DNA damage, induces activation of the tumor suppressor TP53. TP53 activates the expression of genes to aid in stress adaptation. One component of the TP53-mediated transcriptional response is the activation of autophagy (Figure 1).

Indeed, TP53 is positively and negatively involved in the regulation of autophagy [104], and several mechanisms that function in the TP53-dependent autophagy initiation have been described [105]. In particular, TP53 can activate autophagy through the induction of Sestrin-1 and -2 [106,107], unc-51-like kinase 1 (ULK1) [108] or DRAM-1 expression. Sestrin-1 and -2 are involved in AMPK phosphorylation, which, in turn, phosphorylates and activates the TSC1-TSC2 complex, and consequently inhibits the signaling of mammalian target of rapamycin (mTOR), one key autophagy inhibitor [106]. The serine/threonine protein kinase ULK-1, a major component of the ULK complex, which also includes ULK-2 autophagy-related gene (ATG)-101, ATG-13 and FIP200, drives the formation of the initial autophagosomal precursor membrane structure, also called phagophore [109]. On the other hand, several studies demonstrated that TP53 stimulates anti-autophagic responses. In particular, wildtype TP53 proteins localized in the cytoplasm display a negative effect on autophagy [110,111] as gain-of-function mutant TP53 proteins which counteract the formation of autophagosomes and their fusion with lysosomes [112,113].

Autophagy is, thus, a double-edged sword. On the one hand it can protect cells from apoptotic cell death and, on the other hand, it can promote apoptosis depending on several factors including cell type, extracellular nutrient supply, intracellular metabolic activity, and triggering stimuli. Importantly, as mentioned earlier, autophagy contributes to tumor cell resistance to immune killer cells; however, autophagy can also promote T cell or NK cell induced apoptosis. For example, we have recently shown that, through the induction of autophagy, mutant TP53 protein-reactivation increases GzmB- and NK cell-mediated killing of breast tumor cells harboring a mutated TP53 [114]. Indeed, we showed that CP-31398 (a TP53-reactivating small molecule) potentiates NK- and GzmB-mediated lysis by promoting the TP53-dependent induction of Sestrin-1 and -2 and ULK-1 expression, resulting in AMPK activation and mTOR inhibition and autophagosome formation. Moreover, CP-31398-induced autophagy facilitates GzmB- and NK cell-induced mitochondrial outer membrane permeabilization (MOMP) and caspase-3 cleavage in target cells though the selective autophagosomal sequestration of several anti-apoptotic proteins (including Bcl-XL, Bcl-2 and XIAP). Consequently, this study serves to paint a more complex image of how distinct autophagy induction and regulation mechanisms impact the anti-tumor cytotoxic immune response.

## 4. Hypoxia Role in Linking Autophagy and Cancer Stem Cells

The effect of hypoxic stress triggers downstream pathways that further feed and support tumor cell survival, aggressive behavior, and resistance to therapy. Cancer cells undergo adaptive changes in response to hypoxia including EMT. In turn, EMT promotes cancer stem cell (CSC) development. Autophagy has been hypothesized to be exploited by CSCs as an adaptive mechanism to further sustain their survival (Figure 2). The complex pathways involved in EMT, cancer cell stemness and autophagy are interconnected, and are reversible. A trigger in the microenvironment can tip the balance in one direction or the other challenging the cancer cells plasticity and response to environmental stresses.

Hypoxia in the tumor microenvironment is a main feature of solid malignancies. HIF-1α stabilized by hypoxia upregulates several genes, that induce EMT and CSCs, to promote survival in low-oxygen conditions. Hypoxic stress induces the induction of EMT transcription factors SNAI1, SNAI2, TWIST1, and ZEB2 [115]. SNAIL, TWIST and ZEB2 are direct targets of HIF1α [116,117,118,119]; in addition, other master regulators have been shown to mediate EMT processes downstream of hypoxia; these include TGFβ, STAT3, NOTCH and NANOG [120,121]. EMT is a dynamic process that feeds into the CSCs induction and maintenance pathways. Although CSCs account for only a small part of the tumor bulk, they are assumed to be the main players involved in therapeutic resistance, cancer relapse, and distant metastasis. HIF modulates, directly or indirectly, the expression of genes involved in the initiation and maintenance of CSCs including OCT4, SOX2, KLF4, MYC, NANOG, CRIPTO, as *POU5F1,* ALDH1A1, WNT and NOTCH [120,122,123].

Autophagy pathways rely on several factors including the type of stimulus, the cell type, and the microenvironment. Hypoxia contributes to cell survival through the induction of autophagy [124,125]. The link between autophagy, hypoxia and CSCs is attributed to specific proteins that are working together in response to the hypoxic trigger. Specifically, BNIP3/BNIP3L are HIF1α target genes that mediate the induction of autophagy under hypoxic conditions, leading to cell survival [34]. Furthermore, the transcription factor NANOG induced by hypoxia also binds to the promoter element of BNIP3L and induces its expression [126].

Hypoxia inhibits signaling downstream of the PI3K/Akt/mTOR [127]; on the other hand, mTOR is found to interact with and regulate HIF-1α [128,129,130,131]. Inhibiting mTOR was found to reduce the viability of CD133+ pancreatic cancer cells [132] but also causes an increase in CD133+ gastrointestinal cancer cells [131]; in this context, however, HIF-1α induction down-regulated CD133 expression. Anti-cancer drug Gigantol is found to target CSC via suppression of the PI3K/AKT/mTOR and JAK/STAT pathway in lung cancer cells [133]. There is evidence that hypoxia can also activate mTOR in glioblastoma cell lines [134]; upon activation mTORC1 binds to and phosphorylates ATG13 and ULK1 (as part of the ULK1 complex), [135,136] upstream of the Beclin/PI3K complex [137]. In contrast Redd1, a negative regulator of mTOR increases in response to hypoxia; this happens through the action of miR-7 that acts as a repressor of REDD1 and is downregulated under hypoxic conditions [138]. Finally, KLF5, a transcription factor associated with cancer tumorigenicity, increases under hypoxic conditions, and interacts with, and is regulated by, HIF1α [139]. Knock down of KLF5 suppresses the resistance to anti-cancer cisplatin in lung cancer cells, through inactivation of the PI3K/Akt/mTOR pathway [140]. Hence, the signaling pathways involving mTOR and HIF-1α are cell context dependent.

## 5. Key Signaling Pathways Impacted by Autophagy in CSCs

Autophagy pathways are required for maintaining mesenchymal properties. Indeed, inhibiting autophagy in mesenchymal like breast CSCs (BCSCs) results in the re-emergence of epithelial features with a concomitant reduction in CSCs [141]. Several proteins with known functions in autophagy, including ATG4, Beclin and P62 have emerging roles in EMT and/or CSCs maintenance. Overexpression of ATG4A promotes autophagy and proliferation via the AMPK pathway [142]. High expression of ATG4A is associated with poor overall survival of breast cancer patients [143]. Consistent with this, ATG4A promotes the metastasis of gastric cancer cells in vivo and EMT in osteosarcoma [144,145] via the Notch signaling pathway. Interestingly, inhibition of Notch signaling induces autophagy via the (PTEN)-PI3K/AKT/mTOR pathway as well [146]. ATG4A positively impacts CSCs as it promotes gastric CSC-like properties, maintenance, tumorigenicity and the EMT phenotype [147]. Furthermore, ATG4A overexpression induces the expression of the CSCs key genes, Sox-2, Oct-4 and Bmi-1, in gastric cancer cells [145]. Therefore, ATG4A plays a positive role in several types of cancer cell survival and targeting it may prove beneficial.

Beclin1 is necessary for autophagy induction and is involved in EMT. Tumorigenicity of breast CSCs is dependent on Beclin1 and autophagy [148], whereas knockdown of Beclin1 impairs EMT in colon cancer cell lines [149], but also results in activation of STAT3 signaling in CRCs independently of its effects on autophagy [150]. STAT3 is an important regulator of CSCs [151], and its nuclear localization induces autophagy [152]. Inhibiting STAT3 reduces autophagy and tumor growth in the context of acute myeloid leukemia (AML) both in vivo and in vitro [153]. Together, these results indicate that Beclin1 levels modulate CSCs and autophagy through alternative pathways and targeting Beclin 1 may be effective in specific cancer types and stages. P62 functions to activate Beclin1 and autophagy, but it also can function as a negative regulator of autophagy, as is demonstrated by the increase in autophagy following P62 knockdown in various cancer cell lines [154]. P62 expression is increased in BCSCs [155]; here, P62 inhibits the tumor-initiating frequency, as well as the growth rate of BCSC-derived tumor xenografts in immunodeficient mice. However, the role of P62 in the tumorigenicity is independent of its role in autophagy pathways. This could be also due to CD44 expression that results in an increase in P62 and subsequent NRF2 activation, leading to the activation of antioxidant response genes [156]. Knock down of P62 and DRAM1 in mesenchymal glioblastoma tumors results in a decrease in aggressiveness and invasion of the glioblastoma stem cells [157]. Thus, targeting P62 may be effective in some cancers, as demonstrated by the inhibition of P62 in bladder cancer that increases the sensitivity to the anti-cancer drug NVP-BEZ235, which also functions as an inhibitor PI3K/mTOR [158].

## 6. Autophagy and Promotion of Inflammation

The link between autophagy and secretion of inflammatory cytokines was shown when secreted factors from autophagy high and low melanoma cells were compared. It was found that high autophagic activity was positively related to the increased protumor cytokine production such as IL-1β, CXCL8 and LIF (Figure 3). Induction of autophagy in the autophagy low melanoma cells led to increase in the secretion of protumor cytokines and inhibition of autophagy in the autophagy high melanoma cells led to decrease in the secretion of protumor cytokines [159]. In head and neck squamous cell carcinoma (HNSCC), the cancer associated fibroblasts (CAFs) exhibit increased autophagy, which helps in the progression of the disease. The authors show that CAFs in HNSCC show increased secretion of IL6 and IL8 cytokines [160]. Another study used the co-culture model system in which cancer cells and fibroblasts were cultured together; this led to the induction of autophagy in the fibroblasts. Induction of autophagy upon co culture causes the secretion of various protumor cytokines such as: IL-6, IL-8, IL-10, MIp1α, IFNγ, RANTES (CCL5) and GMCSF [161]. Similarly, pancreatic satellite cells (PSCs) undergo autophagy and produce IL-6 cytokine to promote pancreatic tumor growth, and the inhibition of autophagy in PSCs reduced IL-6 and decreased invasiveness [162]. Another proinflammatory cytokine, HMGB1, has been shown to be secreted from glioblastoma and hepatoma cells in an autophagy dependent manner [163,164]. In response to chemotherapy, tumor cells release ATP, which causes the recruitment of dendritic cells and T lymphocytes in the tumor bed. It was reported that the ATP release was mediated by autophagy and inhibiting autophagy led to decreased ATP and recruitment of immune cells [165]. In conclusion, autophagy promotes the secretion of various proinflammatory factors and most of these factors have been shown to promote tumorigenesis, survival, metastasis, and proliferation [166,167,168,169,170,171,172,173,174,175,176].

## 7. Targeting Autophagy to Improve Current Cutting-Edge Therapy Approaches

Advancements in the understanding of interactive tumorigenesis signaling pathways from in vitro and in vivo studies have changed the understanding of tumor therapies. More specific and directed interventions have been developed, or are under development, to regulate apoptotic sensitivity of tumor cells, contributing to the large number of compounds produced, but few of them that had showed successful results in vivo, as was observed in vitro. A plausible explanation is the interference of the tumor microenvironment with the targeted signaling.

Because of the role of autophagy in pathways such as inflammation, apoptosis regulation and EMT/CSC maintenance, targeting autophagy could be a valuable tool in targeting cancer cells. Autophagy has been recently targeted as an anti-tumor therapy due to its assumed pro-survival mechanism. Thus, inhibiting autophagy was thought to help in reducing tumorigenesis. However, recent data are conflicting showing that the therapeutic outcome can be correlated with the significant different and opposite function of autophagy which can be tumorigenic and tumor suppressive depending on type of cells and the proliferation stage. In fact, in healthy cells or in early pre-malignant cells, autophagy can be protective by eliminating transformed cells; however, in late advanced cancer autophagy induction would result in tumor cell survival and proliferation. Although, at this stage, inhibiting autophagy would be considered as a successful target to eliminate tumor cells, studies showed that these interventions should be dealt with precaution as autophagy targeting is more complex even within the same cell population [177,178]. Many clinical trials are ongoing to study the impact of autophagy inhibition (using Chloroquine and its derivative Hydroxychloroquine) on tumor cells such as advanced solid tumors, breast cancers, glioblastoma multiforme, hepatocellular carcinoma, colorectal cancer and others [179]. These drugs were used in combination with other chemo-agents targeting cell cytoskeleton, DNA, kinase inhibitors other metabolic pathways [180]. Currently, thoughts are directed toward targeting specific autophagy components than the general autophagy pathway for better therapeutic outcomes. Ongoing preclinical studies are testing compounds that target more selectively autophagy such as inhibitors for ULK1 and ATG4B [181,182]. Better understanding of the selective autophagy machinery and its interaction with other signaling pathways that influence the survival- and proliferation-related decision of cells is crucial to enable the development of more specific and targeted therapies. Moreover, activation of cell death or inhibition cell proliferation both become effective tools use along with drugs in combination with immunotherapy for targeting autophagy. However, the induction of autophagy may help the tumor cells escape from immune surveillance and further result in resistance to anti-tumor immunotherapy; for effective tumor eradication, it is, therefore, key to determine whether, when and how autophagy can be modulated to target cancer growth. Natural and synthetic compounds that target early or late pathways, proteasomal inhibitors, protein kinase inhibitors and cytotoxic agents exist, are tested, and have been effective in sensitizing cancer cells [183]. In addition, several clinical trials are being conducted to gauge the in vivo effects of these drugs [184]. As CSCs are linked to resistance to therapy, recurrence, and aggressive tumor behavior, understanding the mechanisms underlying the maintenance of CSCs is vital for the development of new therapeutic strategies that target specific populations of CSCs. Indeed, inhibiting autophagy in CRC does reverse the hypoxia-induced phenotype in CSCs [185]. Combinatorial therapy such as radiotherapy in the form of carbon-ion beam treatment combined with 5-FU resulted in increased autophagy and apoptosis in CRC cells including CSCs [186]. This data could indicate that activating autophagy alone may not be sufficient to trigger cell death; in combination with other forms of therapy, however, it will tip homeostatic mechanisms towards cell death.

## 8. Conclusions

Autophagy provides an excellent intracellular recycling system that provides cellular defense, and exploiting autophagy may contribute significantly to disease management and prevention. The tumor microenvironment (TME) is hypoxic, metabolically demanding, and inflammatory. All these factors are known to be conducive for the induction of autophagy. In turn, autophagy orchestrates cancer cell plasticity and impacts critical functions for cellular vitality and organismal homeostasis like apoptosis, oxidative stress response, signaling inflammation, and response to immune cell mediated recognition and killing. Today’s most urgent challenge lies in understanding the mechanisms of autophagy underlying both its suppressor and driver of tumorigenesis through tuning survival in a context dependent manner and in the context of the heterogenous and complex tumor microenvironment. A better appreciation of the complex role of autophagy in the immune-oncology field is particularly crucial. Thus, monitoring autophagy in the tumor microenvironment using in vivo studies will have additional value because this will permit the understanding of all interconnected mechanisms. High-dimensional single-cell studies will allow the study of cellular heterogeneity, analysis of cell identity and will permit monitoring of the evolution of cellular behavior such as metastasis and the effects of treatments; this tool will be highly valuable in optimizing cancer treatment. Although several studies have indeed addressed autophagy in human patients or via knock-down experiments in animal models, it remains uncertain as to when, and under which exact conditions, autophagy is pro-survival or a cell death inducer, and which signals are key during this process. In most cases, it is also not known if the effect of targeting components of autophagic machinery is due to signaling abrogation or to the disrupted autophagic flux per se. Furthermore, it is important not to forget that autophagy also plays key roles in other diseases including obesity and diabetes, cardiovascular disease, neuronal health, bacterial infections, and viral disease [187]. Hence, targeting autophagy to maintain homeostasis needs to be used in conjunction with targeted therapies and, as such, has potential benefit in cancer treatment. It would, indeed, be of major interest to improve our current knowledge on the resultant positive and negative effects of targeting autophagy to positively impact anti-tumor immunity and, thus, resolve several issues that help determine the potential combinatorial use of autophagy inhibitors with immunotherapies in clinical settings.
